# Effect of Yoga and Mindfulness Intervention on Symptoms of Anxiety and Depression in Young Adolescents Attending Middle School: A Pragmatic Community-Based Cluster Randomized Controlled Trial in a Racially Diverse Urban Setting

**DOI:** 10.3390/ijerph191912076

**Published:** 2022-09-24

**Authors:** Alessandra N. Bazzano, Yaoyao Sun, Vaughne Chavez-Gray, Temitope Akintimehin, Jeanette Gustat, Denise Barrera, Cody Roi

**Affiliations:** 1 Center of Excellence in Maternal Child Health, Department of Social, Behavioral and Population Sciences, Tulane University School of Public Health and Tropical Medicine, New Orleans, LA 70112, USA; 2 Institute of Mental Health, Peking University Sixth Hospital, Beijing 100191, China; 3 Department of Epidemiology, Tulane University School of Public Health and Tropical Medicine, New Orleans, LA 70112, USA; 4 Department of Child and Adolescent Psychiatry, Louisiana State University Health Sciences Center, New Orleans, LA 70112, USA

**Keywords:** child, adolescent, mental health, psychology, mind-body therapies, school health services

## Abstract

Mental health conditions in childhood and adolescence are increasing in the U.S. population and require early intervention, as highlighted by a recent Surgeon General’s Advisory on Protecting Youth Mental Health. These health issues, which have been exacerbated by the COVID-19 pandemic, impair functioning, and may lead to longer term reductions in quality of life. Young adolescents are likely to experience stressors including academic pressure, feelings of loneliness and isolation, and excessive exposure to social media, all of which have been made worse by the pandemic and associated disruptions. Universal preventive programs at school serve as an important strategy for equipping youth with coping skills to address current and future social and emotional challenges. Yoga and mindfulness programs have emerged as a promising preventive approach for schools and have proven feasible and acceptable. The current study evaluated a universal, school-based mindfulness and yoga program among youth aged 11–14 in a racially diverse, urban setting in the United States. Outcomes of interest included symptoms of anxiety and depression. Anxiety and depression symptoms decreased in the intervention group, although these differences were not statistically significant. In the control group, anxiety symptoms decreased but depression symptoms increased. The resulting time effect indicated a significant decrease in anxiety symptoms, while the time by group effect revealed a strong trend in depression symptoms. Future research should investigate the utility of yoga and mindfulness interventions for early adolescents in a larger population, and the differences in intervention effect among subgroups, with attention to longer term outcomes.

## 1. Introduction

Anxiety and depression have become increasingly common among youth in the United States over the last decade. Estimates from a pre-pandemic national survey indicated that among children aged 3–17 years, 7.1% had current anxiety problems, 7.4% had a current behavioral/conduct problem, and 3.2% had current depression, with prevalence of each disorder being higher at older ages [1]. The lifetime prevalence of mental disorders in U.S. adolescents is high—with approximately one in every 4–5 youth in the U.S. meeting criteria for a mental disorder with the potential for severe impairment throughout life, including poor academic performance, school absenteeism, and other negative health outcomes over the life course [2]. These figures, from prior to the COVID-19 pandemic, represented a steep increase over previous comparable years, and highlight the preexisting severe gap in addressing problems among youth [3].

Following widespread school closures and the extreme disruption caused by the pandemic, mental health issues have markedly increased among youth and their families [4,5]. In addition, higher levels of screen use have been associated with worse mental health of children and youth during the pandemic [6]. Social media use, economic instability, mortality, and morbidity due to COVID-19, anti-black racism, climate change, and political strife also contribute to mental health concerns in this age group [7]. However, despite increased stressors in recent years, more than 40% of children with anxiety in the United States reportedly receive no treatment [1]. This gap in access to mental health care [8] represents a considerable public health problem given the chronic, relapsing nature of anxiety and depressive disorders, and their debilitating personal, social, and economic burdens over the life course [9,10].

Early intervention in addressing youth mental health issues has been emphasized as an important strategy by policymakers and researchers alike. Merikangas and colleagues highlighted the importance of transitioning the common emphasis on treatment of mental health disorders in youth to focus on prevention and early intervention [2]. Unaddressed behavioral health problems may lead to self-injurious thoughts and behaviors. Suicide is one of the leading causes of death for adolescents and is often but not always associated with pre-existing anxiety and depression [11,12,13]. Parents and caregivers may not be aware of the mental health concerns of children in the course of their normal daily routines, including thoughts of self-harm or suicidal ideation [14,15]. More than two thirds of suicide deaths in adolescence and young adulthood have occurred with no previous suicidal behavior, highlighting the urgent need for early interventions among the general population of young people to address social and emotional health [16].

Yoga and mindfulness provided through school-based programming may provide an important opportunity to address social emotional needs of children, and to provide coping skills, for those who do not receive attention to their behavioral or mental health through other channels. These programs have become more widespread over the last decade in the United States [17]. An emerging evidence base justifies interventions utilizing yoga and mindfulness programs (YMP) in the youth population to improve social and emotional outcomes from early childhood [18] through adolescence [19], including evidence from randomized controlled trials [20,21,22]. A systematic review of utilizing yoga to improve anxiety and depression in youth found that, among studies assessing both anxiety and depression, 58% of reviewed studies identified reductions in both conditions, while 70% of studies assessing anxiety alone showed improvements, and 40% of studies assessing only depression showed improvements [23]. Yoga has also been found to improve youth resilience [24], and may enhance coping skills that could bolster young people’s mental quality of life. Lazarus’ and Folkman’s stress and coping theory provides insight into the potential for coping as an explanatory mechanism, where they describe it as a process by which people utilize cognitive and behavioral efforts in managing stressful situations [25]. In this way, coping is seen as a psychological process focused on persons’ own interpretations of their situation [26].

Children from low-income families are also less likely to be diagnosed and treated for mental disorders [27], putting them at a further disadvantage given evidence demonstrating the impact of poverty on health development and outcomes [28]. Disparities in access to psychotherapeutic and psychiatric interventions in youth are also documented among children from African American and Latinx families, as well as immigrant families, where youth are significantly less likely to receive needed health interventions [29]. Given the potential for school-based interventions to address social and emotional health of young adolescents, and particularly symptoms of anxiety and depression, further evidence on the potential of feasible interventions to improve these is needed.

The current study, conducted in partnership with a middle school in a racially diverse, urban population, located in the southern United States, with high poverty rates among children, sought to evaluate the utility of YMP through a brief, school-based intervention for symptoms of anxiety and depression among youth 11–14 years of age.

## 2. Methods

The study was a pragmatic, single-site, cluster randomized controlled design to evaluate YMP programming for the school setting on symptoms of anxiety and depression among students. CONSORT [30] guidance was used during the design and reporting of the study. The study took place between September 2018 and March 2019, in a middle school located in New Orleans, LA, USA. The research team partnered with school administrators to support the study which assessed a school-based yoga and mindfulness program delivered by a non-profit community partner organization specialized in providing yoga to schools and educators. A waitlist control design was used wherein all students could receive the intervention at the end of the trial period. The school partner provided deidentified data collected by the school to be analyzed in this study in order to evaluate the effect of yoga and mindfulness intervention for symptoms of anxiety and depression among middle school students. The non-profit organization provided all yoga programming using the Yoga Ed curriculum™ through an established relationship with the school prior to the study. The curriculum included both yoga (movement) and mindfulness (awareness) strategies in an integrated offering. Student assent and parental consent were obtained by the school for study participation. The study proposal was reviewed and approved by Tulane University Human Subjects Protection Office Internal Review Board (Reference#2019549). Trial Registration: The study is registered through ISRCTN16686557.

**Figure 1:**
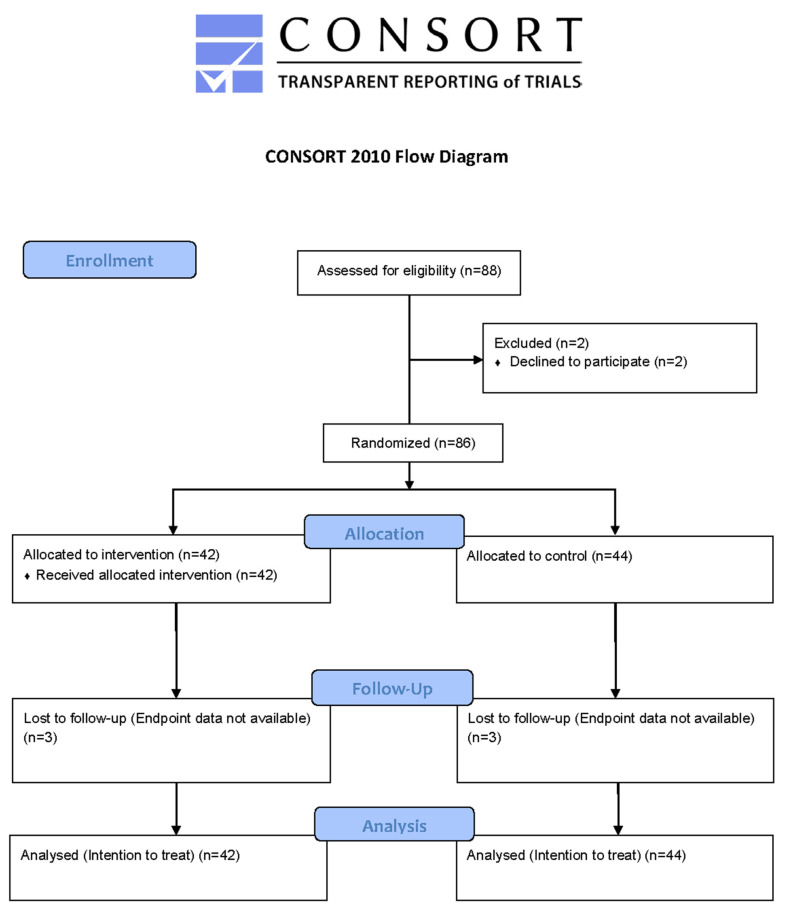
Study flow chart (CONSORT).

Figure 1 below illustrates the study flow chart. Eight groups, or clusters, of students consisting of the entire middle school student population (which included 7th and 8th grades) comprised the participant pool; inclusion criteria were attending the middle school and being willing to participate. The only exclusion criteria were lack of assent and consent to participate for any reason whatsoever (including physical reasons or simple preference). Students aged 11–14 years old were assigned by school administrators into 8 groups of 10–12 students each, based on their homeroom and weekly course schedules. Four groups received the intervention and four groups acted as controls, receiving the yoga programming only after the study concluded. Randomization was used to determine the order of the group start to intervention condition. Eighty-six of 88 students assented and were consented to participate. At the termination of the intervention study, all 88 students of the middle school participated in programming which was organized by the existing community partner organization and took place in homeroom class.

The CONSORT flow chart is illustrated below in Figure 1.

Students assigned to the intervention group participated in an 8-week curriculum of yoga programming, with sessions once during a school week, each lasting 45 min. Yoga and mindfulness intervention was provided to each student in the intervention group using the Yoga Ed curriculum, which has been previously studied [20,31,32]. The curriculum (with which the study authors have no affiliation) is described as an evidence-based educational program aiming to promote children’s and teens’ health and wellness through the practice of yoga in a school-based environment. During the program, basic skills including breathing exercises, yoga postures, games, and relaxation were practiced in the classroom. A standard intervention session included the following segments: focusing and breathing exercises (about 5 min), warm-up (about 10 min), yoga postures (about 20 min), games and instructional content (about 5 min), and final relaxation (about 5 min). Warrior poses, triangle pose, sun salutations, twists, and child poses were among the positions that were often done. As the curriculum developed, each session added to what came before and introduced new postures. Examples of breathing exercises were deep breathing with a focus on the exhale, and gentle breathing. Activities encouraging the use of yoga poses or breathing in order to mitigate relating stressful life events and social engagement were frequently encouraged through the classes, for example with peer-to-peer listening and discussions. Two yoga teachers led the intervention groups, both of whom were experienced children’s yoga teachers and had undergone the same training on the curriculum. The waitlist control groups received an attention control, consisting of their course schedule as usual, with classes as usual (e.g., art, French, or study hall). School staff randomized the order of eight groups to receive intervention through an open-source website randomizer.org [33]. Blinding by school staff was not possible in the current study due to real world conditions in the school.

**Figure 2:**
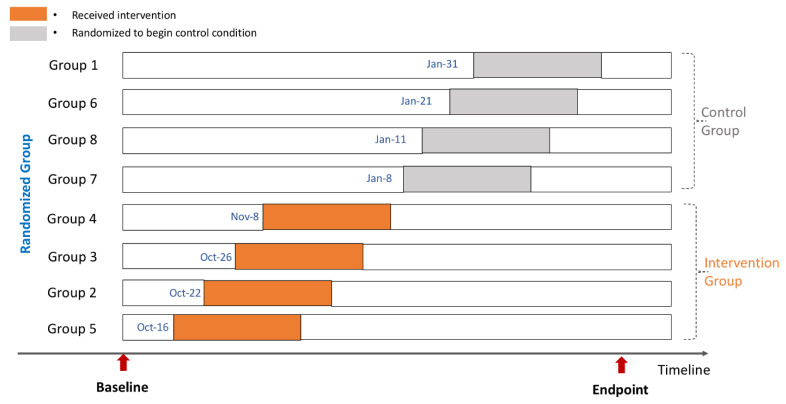
Study data collection.

The Baseline data were collected across all eight groups in September 2018. Eight groups then were randomized to receive control or intervention in October 2018. Final assessments of intervention effect across the eight groups were performed at Endpoint in March 2019, five months after randomization. The study data collection is illustrated in Figure 2 below.

### 2.1. Study Population

The study included the entire student population at a middle school consisting of 88 students. A total of 86 students aged 11–14 years old were assigned, based on their weekly course schedules, into 8 groups of 10–12 students each group; two students declined to participate in the intervention. No adverse events were reported during the course of the study, and the two students who declined to participate did not state their reasons.

De-identified demographic data from school records including gender, race, status of free/reduced price lunch, Individualized Education Program (IEP), and the 504 plan were linked with participant codes allowing for analysis. The IEP is a plan or program developed to ensure that a child who has a disability or is identified as gifted and is attending an elementary or secondary educational institution receives specialized instruction and related services. The 504 Plan is a plan developed to ensure that a child who has a disability identified under the law and is attending an elementary or secondary educational institution receives accommodations that will ensure their academic success and full access to the learning environment. For students with disabilities who do require specialized instruction, the Individuals with Disabilities Education Act (IDEA) controls the procedural requirements, and an IEP is developed. The IDEA process is more involved than that of Section 504 of the Rehabilitation Act (for more information on 504 plans and IEP, please see the United States Department of Education [34].

### 2.2. Instruments

The two main outcome measures for the study included scores from the Patient Health Questionnaire revised for adolescents (PHQA) and the Screen for Child Anxiety Related Disorders (SCARED). PHQA is a 9-item screening measure modified for children ages 11–17 to assess episodes and severity of depressive disorder [35]. Children’s depression symptoms in the past 7 days were self-rated. Each item on the measure is rated on a 4-point scale (0 = Not at all; 1 = Several days; 2 = More than half the days; and 3 = Nearly every day). The total score ranges from 0 to 27, with higher scores indicating greater severity of depression. The Cronbach’s alpha was 0.882 in the current study. The Screen for Child Anxiety Related Disorders (SCARED) is a series of 41 sentences describing how young people feel during the past 3 months, each of which is ranked from 0 (“not true or hardly ever true”) to 2 (“very true or often true”). The total sum score of the 41 items showed a satisfactory internal consistency and discrimination validity in a children sample [36]. The total score of the 20 items on the SCARED screen ranged from 0–40. The Cronbach’s alpha for the 20-item SCARED was 0.899.

### 2.3. Statistical Analyses

SPSS software (version 23.0, Chicago, IL, USA) was used to analyze the data. Analyses were conducted based on an intention-to-treat (ITT) approach, meaning that participants were analyzed in accordance with their initial group assignment. Where PHQA and SCARED items were missed by chance, these were imputed with the mean. All data were summarized with descriptive statistics (mean and standard deviations or frequency and percent). Student’s *t*-test and the chi-square test were performed to examine differences at baseline across groups. Mean differences at Baseline and Endpoint were also calculated and tested in post-hoc analysis. To compare the difference between intervention and control over time, generalized estimated equations (GEE) with an unstructured working correlation matrix and full maximum likelihood estimation were conducted using Baseline and Endline assessments, which showed the main effects of group and time, and also the group by the time interaction effect. GEE was developed for repeated measures data because it could fit data with missing values and considered the within-group correlation. Covariates at baseline included sex (as self-reported in school records), race (as self-reported in school records), free or reduced-price lunch status (as self-reported in school records), IEP status (yes/no), and 504 plan status (yes/no); these were included in adjusted GEE models. Post-hoc statistical power calculated using G*Power on the basis of repeated measures with interaction reached 90% with a total sample size of 76 and effect size f = 0.19 (Cohen’s d = 0.38). This suggests that the sample size of the current study was sufficient to identify any intervention effect. The study is registered through the clinical trial registry ISRCTN under ISRCTN16686557.

## 3. Results

Four groups with a total of 42 students received the intervention (intervention group) and four groups with 44 students received an attention control (control group). Of the 82 students for whom all demographic data were available from school records (some records were not allowed to be linked to protect privacy), 44 (53.7%) identified as male and 38 (46.3%) as female. The racial composition consisted of 55 (67.1%) African American, 19 (23.2%) white, 6 (7.3%) Asian and 2 (2.4%) multi-racial. Most students were non-Hispanic American (80, 97.6%). Nearly half of the sample received free or reduced lunch (40, 48.8%). Regarding the IEP, five students (6.1%) were identified as having a disability, followed by 22 students (26.8%) identified as gifted or talented. The majority of students, 69 (84.1%), reported no 504 plan. As shown in Table 1, Student’s *t*-test and chi-square test suggested no significant differences between intervention and control group at baseline on any demographic characteristics or scores of PHQA and SCARED. The absence of the significance at baseline suggested a successful randomization.

**Figure 3:**
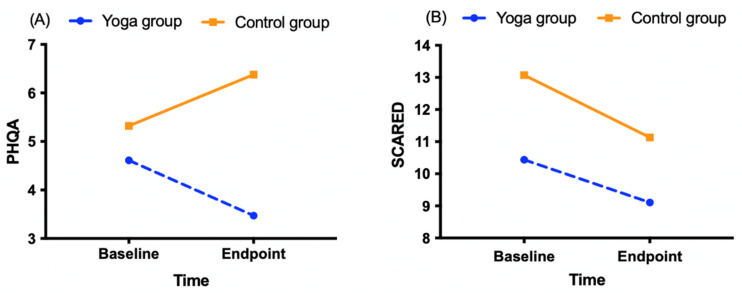
Change in mean scores on PHQA and SCARED scores for intervention and control groups on Baseline and Endpoint. (**A**) PHQA score in total sample; (**B**) SCARED score in total sample.

Means and standard deviations for PHQA and SCARED at Baseline and Endpoint are presented in Table 2 and Figure 3 below. Independent samples’ *t*-test reported significant between-group difference at endpoint on PHQA (3.47 ± 4.37 vs. 6.38 ± 6.997, *p* = 0.037), but no significant difference was found on SCARED.

To assess the intervention effect compared to control group, a GEE model was utilized for PHQA and SCARED including Baseline and Endpoint. The model allows the assumption of data missing at random. We performed sensitivity analyses to assess the robustness of our conclusions and departures from the assumption. The GEE-adjusted model for the intervention effect on PHQA showed no significant main effects by group (Wald *χ*^2^ = 1.852, *p* = 0.174) and time (Wald *χ*^2^ = 0.011, *p* = 0.918), but a trend for the group by time interaction effect (Wald *χ*^2^ = 3.695, *p* = 0.055). The results for SCARED in a GEE-adjusted model indicated a significant main time effect (Wald *χ*^2^ = 8.77, *p* = 0.003), and details are shown in Table 3.

The effect sizes were 0.38 and 0.07 for PHQA and SCARED, respectively. Post-hoc statistical power calculated using G*Power on the basis of repeated measures with interaction reached 90% with a sample size of 76.

## 4. Discussion

This pragmatic trial evaluated a school-based yoga and mindfulness program in a real-world context. Anxiety and depression symptoms decreased in the intervention group, although these differences were not statistically significant. In the control group, anxiety symptoms decreased but depression symptoms increased. The resulting time effect indicated a significant decrease in anxiety symptoms, while the time by group effect revealed a strong trend in depression symptoms.

School based approaches to addressing stress and social emotional needs of youth have been increasingly studied [37,38]; however, few have focused specifically on middle school aged children (11–14 years of age) who may have distinct needs from older adolescents [39] and younger children. The current study provides new evidence for utilizing YMP with middle school students, where previous studies have focused on elementary school children [17,40,41] or high school students [31].

YMP may be particularly well suited to addressing the social emotional needs of young people in school-based settings. Among systematic reviews of yoga for health outcomes in youth, findings have been positive [17,23,40,42,43] notwithstanding challenges in heterogeneity among studies and low to moderate quality of evidence. This study also represents an important addition to the sparse literature from urban settings on school-based yoga for youth indicating the potential for feasibility and effectiveness [41,44].

School-based universal programming for mental health [45], such as YMP employed in the current study, has a number of advantages as an approach to prevention and mitigation of behavioral health morbidity among young people. Providing programming at school partly relieves the burden of accessing care for parents/guardians, many of whom have day to day stressors of their own, and makes programming widely available to a range of students [46]. In addition, such universal programs provided to all students within a school are likely to be less stigmatizing compared with targeted programs, as students need not be selected on the basis of existing problems for inclusion [47]. In populations such as the deep South, where poverty and social determinants of health create grave disparities in access to care, general population programs aimed at preventing or improving behavioral health problems may surmount many of the challenges to access rooted in such disparities (long wait times to see providers, expenses associated with treatment, transportation to providers, and time commitment by caregivers who may be working multiple jobs) [48].

Middle-to-late adolescence is characterized by problems of emotional control, and biological changes taking place during puberty may undermine the ability to cope with stress and give rise to unhealthy risk-taking behavior [49]. By intervening earlier in adolescence, through universal social and emotional care programs such as school based YMP, youth may become better equipped with coping skills and techniques to combat stress and mitigate negative emotional states to protect future health and developmental trajectory.

Strengths of the study include real-world rigor in a community-based setting (including randomization and counterfactual) and developmentally appropriate, validated, and reliable outcome measures for symptoms of anxiety and depression. The study clearly described evidence-informed intervention modalities including physical yoga postures, breathing, meditation, and relaxation. As the programming was tailored specifically for the school’s needs, the delivery method, timing, and setting can be seen to be optimally appropriate to the needs of students. No adverse events were reported during the course of the study and only two students declined to participate for unstated reasons.

The study had several limitations, which require that results be interpreted with caution. Sample size was limited, and small between-group mean differences were reported, suggesting the need to be cautious in extrapolating the intervention effect. As in any study, missing data had to be accounted for in statistical analysis. The time sequence (treatment groups, control groups) design may lead to unbalanced maturation effects and history. Access to yoga trainers could limit future reproducibility of results and application of the specific method. Finally, though no reports of excessive absences were received, limited information related to attendance in the intervention was available to allow evaluation of fidelity.

Future studies could investigate which factors may be responsible for time-related decrease of anxiety symptoms among middle school children in such programs. These could be related to the timing of assessments or other school related events. Another worthy topic of investigation may be why YMP did not help in reducing internalization symptomatology. The inability of the study results to establish a significant impact of YMP on symptoms may be attributable to constraints of real-world application of the intervention. Future research on effects of yoga and mindfulness programs in youth are warranted, particularly in light of the disruptions caused by the COVID-19 pandemic; however, larger scale studies would be required to robustly assess the long-term effect of school-based yoga and mindfulness programs.

## 5. Conclusions

While this study was unable to provide definitive results regarding reduction of symptoms of anxiety and depression among youth aged 11–14 years old in a diverse, urban setting, further studies are warranted. Future studies may be better able to determine the conditions for effective use of YMP, including which subgroups of youth may benefit from mind-body therapies, and whether improvements are lasting over time.

## Figures and Tables

**Table 1:** Demographics and baseline variables of intervention and control group.

Variable	Total	Intervention Group	Control Group	*t*/*χ*^2^	*p*-Value
Sex	(*n* = 82)				
Male	44 (53.7%)	23 (57.5%)	21 (50.0%)	0.463	0.50
Female	38 (46.3%)	17 (42.5%)	21 (50.0%)		
Race	(*n* = 82)			3.403	0.32
Asian	6 (7.3%)	3 (7.5%)	3 (7.1%)		
Af. Am.	55 (67.1%)	30 (75.0%)	25 (59.5%)		
White	19 (23.2%)	7 (17.5%)	12 (28.6%)		
Multi-racial	2 (2.4%)	0	2 (4.8%)		
Ethnicity	(*n* = 82)				1.00
Non-Hispanic	80 (97.6%)	39 (97.5%)	41 (97.6%)		
Eligibility for lunch	(*n* = 82)			1.233	0.27
Full price	42 (51.2%)	23 (57.5%)	19 (45.2%)		
Free or reduced	40 (48.8%)	17 (42.5%)	23 (54.8%)		
IEP	(*n* = 82)			2.347	0.30
Disability	5 (6.1%)	4 (10.0%)	1 (2.4%)		
Gifted/Talented	22 (26.8%)	9 (22.5%)	13 (31.0%)		
No IEP	55 (67.1%)	27 (67.5%)	28 (66.7%)		
504 Plan	(*n* = 82)			0.658	0.42
Yes	13 (15.9%)	5 (12.5%)	8 (19.0%)		
No	69 (84.1%)	35 (87.5%)	34 (81.0%)		
PHQA					
Baseline (*n* = 79)	4.97 ± 5.684	4.61 ± 4.595	5.32 ± 6.574	0.561	0.58
SCARED ^a^					
Baseline (*n* = 82)	11.82 ± 8.303	10.44 ± 7.104	13.07 ± 9.161	1.444	0.15

^a^ sum of items 1–20 of SCARED.

**Table 2:** Means and standard deviations for outcome variables.

	Total	Intervention Group	Control Group	*t*/*χ*^2^	*p*-Value
*PHQA*					
Baseline	4.97 ± 5.684	4.61 ± 4.595	5.32 ± 6.574	0.561	0.58
Endpoint	4.95 ± 5.995	3.47 ± 4.37	6.38 ± 6.997	2.120	0.037 *
*SCARED*					
Baseline	11.82 ± 8.303	10.44 ± 7.104	13.07 ± 9.161	1.444	0.15
Endpoint	10.12 ± 8.296	9.11 ± 7.866	11.13 ± 8.690	1.066	0.29

*Note.* * Significance was set at *p* < 0.05.

**Table 3:** Overall test and mean difference between intervention and control groups for the outcomes in GEE analysis.

		MD _CG-IG_	Group Effect		Time Effect		Group × Time Effect	
			Wald *χ*^2^	*p*-Value	Wald *χ*^2^	*p*-Value	Wald *χ*^2^	*p*-Value
PHQA (*n* = 80)	Baseline	1.20 (−1.54, 3.93)	1.852	0.174	0.011	0.918	3.695	0.055
	Endpoint	2.46 (−0.23, 5.14)						
SCARED (*n* = 81)	Baseline	1.98 (−1.51, 5.48)	1.935	0.164	8.77	0.003	0.343	0.558
	Endpoint	2.71 (−0.84, 6.26)						

Note: MD _CG-IG_: mean difference from control group to intervention group; bold text indicates statistically significant result.

## Data Availability

The data presented in this study are available on request from the corresponding author and the partner school. The data are not publicly available due to privacy concerns.
